# Spontaneous Intracranial Hemorrhage in Liver Failure: A Case-Based Approach to Transplant Candidacy and Care

**DOI:** 10.7759/cureus.104651

**Published:** 2026-03-04

**Authors:** Ramona Nicolau Raducu, Eslam Fouda, Nicolas Caram, Elharith AL-Akeel, Vinaya Manmohansingh, Yehuda Raveh, Gisele J Wakim, Carolina Benjamin, Fouad Souki

**Affiliations:** 1 Anesthesiology, Jackson Memorial Hospital, Miami, USA; 2 Radiology, Jackson Memorial Hospital, Miami, USA; 3 Anesthesiology, University of Miami/Jackson Memorial Hospital, Miami, USA; 4 Neurosurgery, Jackson Memorial Hospital, Miami, USA

**Keywords:** acute liver failure, alcohol cirrhosis, liver transplantation, porto-mesenteric venous thrombosis, spontaneous intracranial hemorrhage

## Abstract

Intracranial hemorrhage in patients with acute or chronic liver disease is a life-threatening complication with repercussions on both liver transplantation candidacy and perioperative transplant management. This case series and review of the literature details the perioperative management and outcomes of three patients who experienced spontaneous intracranial hemorrhage and subsequently underwent liver transplantation. These cases highlight the challenges encountered in such complex scenarios, emphasizing the critical need for individualized assessments, tailored management strategies, and a multidisciplinary approach to maximize patient outcomes. Moreover, these cases underscore how evolving neurocritical care practice, advances in hemostatic therapies, and improved coordination between transplant and neurology teams can expand access to transplantation. Collectively, these observations contribute to a growing evidence base, supporting nuanced decision-making in situations traditionally considered prohibitive for transplant surgery.

## Introduction

Spontaneous intracranial hemorrhage (ICH) in patients with end-stage liver disease (ESLD) poses significant challenges, particularly for those awaiting liver transplantation (LT). The incidence of ICH is significantly higher in ESLD compared to the general population, with reported hazard ratios ranging from 1.5 to 2.4 [1-4]. Outcomes are notably poor: survival rate drops to 22% in ESLD patients with ICH, and further declines to 14% among those on the transplant waiting list, compared to approximately 50% in the general population [5-6].

Managing spontaneous ICH in the setting of acute or chronic liver disease requires highly specialized perioperative care. This includes addressing coagulopathy, managing cirrhotic hemodynamics, ensuring timely neurosurgical monitoring, intervention, and navigating the complexities of multisystem organ dysfunction. A central ethical and clinical dilemma arises: should the occurrence of a stroke influence transplant candidacy? On one side, LT may offer substantial benefits, potentially reversing coagulopathy, improving metabolic function and enhancing neurological recovery. On the other hand, the presence of ICH may signal a poor neurological prognosis, raising concerns about post-transplant outcomes and the ethical allocation of scarce donor organs under the principle of distributive justice.

The aim of this study is to describe perioperative management of LT in patients with spontaneous ICH, to examine how the presence of ICH influenced transplant candidacy decisions, and to summarize our institution's practical recommendations for clinical practice. This case series highlights the critical importance of a coordinated, multidisciplinary approach involving transplant surgeons, anesthesiologists, intensivists, neurosurgeons and other specialists. Such collaboration is essential to navigate the delicate balance between the neurological risk and the lifesaving potential of LT.

## Case presentation

We present three consecutive single-center cases in which a spontaneous ICH preceded LT. All cases were inpatient encounters and were identified from our transplant database between 2021 and 2023.

Case one

**Figure 1:**
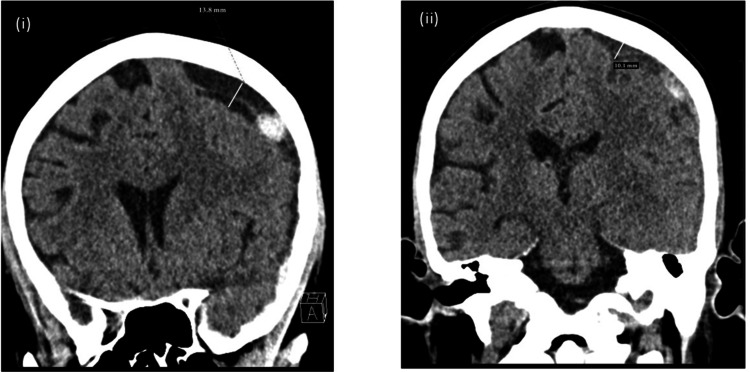
Pre and post-transplant imaging (i) Pre-transplant: CT brain shows a left hemispheric subdural collection with mixed fluid and hyperdense hemorrhagic contents, measuring up to 14 mm in thickness, extending to the left posterior parafalcine region and overlying the left tentorial leaflet, with a slight mild left uncal medialization and up to a 3 mm left-to-right midline shift. (ii) Post-transplant (Day 0): CT brain shows an evolving left hemispheric subdural hematoma with slightly decreased density areas, measuring approximately 10 mm in width and a midline shift of 2 mm.

A 34-year-old man with decompensated alcohol liver disease and anxiety disorder was transferred for LT. He was admitted to the intensive care unit (ICU) with a Model for End-Stage Liver Disease (MELD) score of 41, suffering from mild encephalopathy, jaundice, abdominal distension, and on renal replacement therapy (RRT). On hospital day three, his mental status acutely worsened. Although he continued to follow commands, neurologic examination revealed dysarthria and decreased strength in the right upper and lower extremities. A brain computerized tomography (CT) scan revealed a significant left hemispheric acute subdural hemorrhage with midline shift and a smaller right frontal subdural haemorrhage (Figure 1 (i)).

He was intubated on day four for airway protection. The neurosurgical protocol in our institution targeted correction of coagulopathy with the goal of an international normalized ratio (INR) < 1.4 and a platelet count > 100,000/μL, along with hypertonic saline to maintain serum sodium of 140-145 mEq/L and initiation of levetiracetam. Despite transfusions of blood products, the INR remained between 1.6-1.8 and platelet counts persisted at 20-29 x10^3^/μL, while fibrinogen levels were normal (200-400 mg/dL). Serial follow-up CT scans showed stable ICH.

The patient was listed for LT on day seven and received a liver from a 36-year-old donor on day 15. The surgery, complicated by adhesions/necrotic tissue from chronic pancreatitis, involved a piggyback orthotropic LT with a temporary portocaval shunt and a significant blood loss (7 liters). Intraoperative management included vasopressors and extensive transfusion (total of 16 units packed red blood cells (pRBCs)), 14 units fresh frozen plasma (FFP), three units (10 pool/unit) platelets (PLTS) and one unit (10 pool/unit) cryoprecipitate (CRYO), based on serial thromboelastography (TEG). Two liters were removed via ultrafiltration, while 2 liters of ascites were drained, and 1.4 liters of urine was produced. The surgery lasted 3.9 hours, with warm ischemia time of 21 minutes and cold ischemia time of 5.4 hours. 

Postoperatively, the follow up CT brain show stable subdural hematomas (Figure 1 (ii)). Medical management continued with goals of INR<1.4, platelet count >50,000/μL, normal serum sodium and blood pressure, levetiracetam until post-operative day (POD) 15. The patient underwent a washout procedure on POD two and fascia closure on POD four. Pancreatitis was treated with total parenteral nutrition, enteral nutrition and octreotide. He was extubated on POD seven but reintubated the following day due to lethargy, hypoxemia, and worsening respiratory effort, remaining intubated until POD 15. Kidney function recovered and RRT was stopped on POD 11. The patient developed lower extremity deep venous thrombosis on POD eight, with an inferior vena cava filter placed on POD 12. CT angiography on POD nine showed thrombosis of the right internal jugular vein and dural venous sinus, followed by pulmonary embolism on POD 11. Heparin drip was initiated on POD 11, continued until POD 22, then switched to Lovenox (enoxaparin). Follow up CT brain and neurological exam were unchanged. The patient was discharged from the ICU on POD 22 and transferred for rehabilitation on POD 30. At the three-year follow-up, the patient continued to exhibit no neurological deficits with normal motor exam and speech ability.

Case two

**Figure 2:**
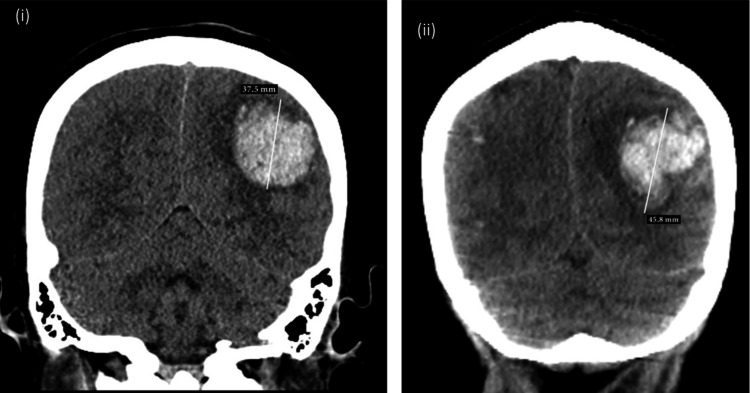
Pre and post-transplant imaging (i) Pre-transplant: CT brain shows intraparenchymal hemorrhage in the left posterior parietal lobe, measuring 3.7 x 2.4 cm, with a mass effect and rightward midline shift of approximately 4.3 mm. (ii) Post-transplant (Day 0): CT brain shows an increase in the size of the largest hemorrhage in the left parietal lobe, now measuring 5 x 3 x 4.5 cm, with increased localized vasogenic edema and a midline shift of 8 mm.

A 45-year-old woman with acute liver failure (ALF) due to recent acetaminophen overuse was transferred for LT. She was intubated, on hemodynamic support, on RRT, on continuous N -acetyl cysteine infusion with a MELD score of 35. She was listed as status 1A, the highest priority category for LT, reserved for patients with ALF who have a life expectancy of only few days without urgent transplantation. Initial neuroimaging was normal; however, a repeat scan on day two, revealed multiple bilateral frontoparietal hemorrhages with edema and midline shift. Her coagulopathy (INR 8.09; fibrinogen 91 mg/dL, platelet count 39 x10^3^/μL) was corrected with plasmapheresis and blood products and seizure prophylaxis was initiated. On day three, a suitable 30-year-old donor became available. Despite correction of her coagulopathy to INR 1.61, fibrinogen 154 mg/dL and platelet count 43 x10^3^/μL, a pre-transplant brain CT scan demonstrated a new left anterior frontal lobe hemorrhage and interval enlargement of the left posterior parietal hemorrhage, with worsening midline shift (Figure 2 (i)).

Given the rapidly progressive ALF and the poor prognosis without transplantation, a multidisciplinary team elected to proceed with the LT despite the elevated neurologic risk.

Intraoperative management included maintaining systolic blood pressure (SBP) >100 mmHg, correcting coagulopathy based on serial TEG and managing intracranial pressure (ICP). Point of care optic nerve sheath ultrasound (ONSUS) was used to measure optic nerve sheath diameter (ONSD) as a non-invasive tool for ICP monitoring. This was done alongside head elevation, maintaining PaCO2 30 mmHg though hyperventilation; administering mannitol 20% at 1 g/kg with ultrafiltration via intraoperative RRT and providing anti-seizure prophylaxis with levetiracetam. The patient underwent a piggyback orthotropic LT with a temporary portocaval shunt. The surgery lasted 4.3 hours, with the donor liver warm ischemia time of 20 minutes and a cold ischemia time of 5.7 hours. The surgery resulted in an estimated blood loss of 5 liters, with an additional 2 liters removed via ultrafiltration. Intraoperatively, the patient received 11 units pRBCs, 10 units FFP, three units PLTS and two units CRYO.

Postoperatively, CT showed worsening ICH, increased edema and midline shift (Figure 2 (ii)), leading to fiberoptic ICP-monitoring bolt insertion on POD zero (initial opening pressure 27 mmHg) and an external ventricular drain (EVD) on POD one. Stabilization of ICH and gradual improvement in cerebral edema and neurological exam lead to removal of the EVD on POD nine. The patient was successfully extubated on POD seven and RRT was stopped on POD 14. Acute partial occlusion of the right internal jugular was diagnosed on POD 18. She was discharged from ICU on POD 21 and from the hospital on POD 30 to acute inpatient rehabilitation for severe proximal muscle weakness. Readmission occurred on postoperative day 32 following new onset generalized seizures, with a CT scan revealing worsening vasogenic brain edema. Remarkably, a two-year follow-up revealed no neurological deficit and a normal motor exam and speech ability.

Case three

**Figure 3:**
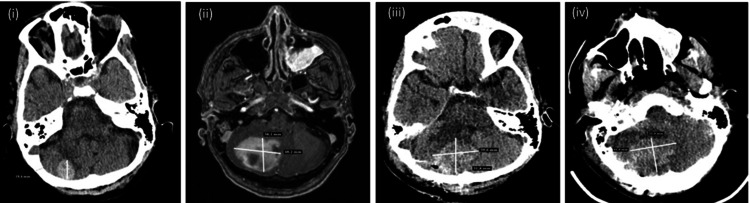
Pre and post-transplant imaging (i) Day 15, Pre-transplant: CT brain shows a 2 cm hyperdense lesion in the right cerebellar hemisphere with neighboring vasogenic edema, suspicious for hemorrhagic metastasis vs hematoma. (ii) Day 30, Pre-transplant: MRI brain shows a significant interval increase in the size of the hemorrhagic right cerebellar lesion, measuring approximately 3.8 x 4.8 x 2.6 cm, with increased surrounding vasogenic edema. (iii) Day 35, Post-craniotomy, Pre-transplant: CT brain shows postsurgical changes of right suboccipital craniotomy with an interval increase in size and density of a right posterior fossa hematoma, measuring approximately 3.1 x 4 x 3.2 cm. (iv) Day 37, Post-transplant: CT brain shows a grossly unchanged posterior fossa hematoma, measuring approximately 3.2 x 4.2 x 3.3 cm.

A 42-year-old woman with ESLD secondary to alcohol and porto-mesenteric vein thrombosis stage three to four, was transferred for LT evaluation with a MELD score of 38. Intubation was required from day four to nine due to worsening lung opacities and hypoxemia. Acute kidney injury secondary to hepato-renal syndrome required RRT until day 15. On day 15, CT brain revealed a right cerebellar lesion hemorrhagic lesion (Figure 3 (i)).

The magnetic resonance imaging (MRI) report confirmed hemorrhagic lesion, and metastatic disease workup was negative, leading to listing for LT. Due to acute metal status changes, on day 30, repeat neuroimaging showed increased lesion size with edema and mass effect (Figure 3 (ii)). A craniotomy was performed on day 31, confirming an organizing blood clot. Despite stable neurological exams, further neuroimaging on day 35 showed increased hematoma size (Figure 3 (iii)).

Liver transplant proceeded on day 37 when a 53-year donor became available. Significant intraoperative challenges occurred, including maintaining hemodynamics, extensive blood products transfusion (29 units pRBCs, 25 units FFP, six units PLTS, two units CRYO) and additional therapies (500 units Prothrombin complex concentrate, 2 g fibrinogen concentrate) used to correct coagulopathy based on TEG/laboratory tests. The blood loss was 15 liters and 2 liters were removed via ultrafiltration. Due to extensive porto-mesenteric vein thrombosis, blood flow was established through an interposition vein graft from the superior mesenteric vein using donor external-common iliac veins, followed by a piggyback LT and fascia was left open. The surgery lasted 7.15 hours with 23 minutes warm ischemia time and 4.9 hours cold ischemia time. The pathology report was consistent with end stage steatohepatitis and well-differentiated hepatocellular carcinoma (stage II).

Postoperatively, brain CT scans showed stable hematoma (Figure 3 (iv)). The patient underwent abdominal washout and fascia closure on POD two, was extubated and RRT continued until POD four. The patient was discharged from the ICU on POD 14. An MRI on POD 19 revealed a small 3 mm cavernous internal carotid artery aneurysm and decreased cerebellar hematoma size. She was transferred to rehabilitation on POD 39. Complications included biliary stricture, pathologic radius fracture, sacral ulcer, and persistent infections. On POD 72, she was diagnosed with acute pulmonary embolism and treated with apixaban. The patient remained free of neurologic deficits at the two-year follow-up with normal motor and speech ability.

## Discussion

This case series underscores the complexity of managing spontaneous ICH in patients with alcohol-related cirrhosis or ALF, particularly in the context of LT.

Spontaneous ICH in cirrhosis and ALF

Spontaneous ICH in patients with cirrhosis, particularly alcohol-related (cases one and three), is a significant clinical concern. Huang et al. found that ICH outcomes in cirrhosis are more closely linked to alcohol-related liver disease than thrombocytopenia or elevated INR [7]. In ALF (case two), abrupt coagulation changes can precipitate ICH, necessitating vigilant monitoring and rapid intervention. Stravitz et al. reported that 11% of patients with ALF experience bleeding complications, with 1% developing ICH, often in the setting of extrahepatic organ failure and low platelet counts rather than elevated INR [8]. 

Coagulopathy management in ESLD

Managing coagulopathy in ESLD is particularly challenging due to the elevated INR, low fibrinogen, and thrombocytopenia. While, preoperative prohemostatic therapy is essential, it may not always prevent ICH progression, as seen in cases two and three. Advanced therapies such as plasma exchange can rapidly improve coagulation and hemodynamic parameters in ALF [9]. Additional agents like fibrinogen concentrate and prothrombin complex concentrate were used intraoperatively in case three to manage coagulopathy [10,11]. The optimal threshold for platelet transfusion remains debated. While levels above 100x10^9^/L are often targeted, some studies suggest that 50x10^9^/L may suffice to preserve thrombin generation in cirrhotic patients [12]. Maintaining fibrinogen levels above 150 mg/dL is recommended for central nervous system bleeding [13]. TEG, used in all cases, provided dynamic and individualized guidance for managing coagulopathy [14].

Blood pressure and ICH risk

Although hypertension is a well-established risk factor for ICH in the general population, it is not typically associated with ICH in ESLD [15]. However, post-transplant SBP management is critical, as normalization of vascular tone can lead to SBP increases of more than 30 mmHg from pre-transplant levels [13,16]

Transplant candidacy and multisystem considerations

Proceeding with LT in patients with spontaneous ICH requires multidisciplinary evaluation. Balancing the risks of intracranial bleeding with the urgency of treating liver failure is essential, especially in patients with high MELD score (>35) or ALF, who face increased risk of both bleeding and mortality without transplantation. All patients in this series required perioperative dialysis, adding further complexity.

Neurological monitoring and imaging

Serial neuroimaging and neurological exam are vital. Intracranial pressure monitoring can be a non-invasive to invasive method. ONSUS is a valuable non-invasive tool: an ONSD of around 5.00 mm or greater is commonly associated with ICP >20 mmHg [17]. In case two, ONSD remained below this threshold, providing reassurance. Fiberoptic ICP monitors are valuable invasive tools for measuring ICP, easier to place, and carry a lower bleeding risk compared with EVD in ALF [18]. EVDs, while more challenging to place due to the need for precise placement within the ventricle, provide reliable drainage of cerebrospinal fluid and accurate pressure measurements [19]. When fiberoptic ICP monitoring failed, transitioning to EVD was critical for effective ICP management.

Surgical intervention

Craniotomy in ESLD is reserved for life-threatening situations due to the high risk of complications, rebleeding, and mortality [20]. However, a recent multicenter randomized trial showed that minimally invasive lobar hematoma evacuation within 24 h improves 180-day functional outcomes compared with medical management [21]. In case three, craniotomy was likely life-saving when combined with aggressive coagulopathy reversal strategies.

Intraoperative techniques and postoperative care

Surgical techniques such as the piggyback method and portocaval shunt were employed to maintain hemodynamic stability during LT, by preserving venous return and reducing portal pressure [22]. Postoperatively, the focus shifts from bleeding to venous thromboembolism (VTE) risk. Platelet count typically reaches a nadir around postoperative days three to five and recovers over two weeks [23]. All three patients developed VTE postoperatively, highlighting the need for individualized prophylaxis. Intermittent pneumatic compression devices should be initiated at ICH diagnosis, and pharmacologic prophylaxis (e.g., unfractionated or low molecular-weight heparin) should be started only after confirming hemorrhage stability via neuroimaging [24].

This case series illustrates that spontaneous ICH in a patient with liver failure undergoing LT demands a multifaceted approach. Key elements include multidisciplinary coordination, thorough risk-benefit analysis, careful preoperative planning, meticulous intraoperative management, advanced surgical techniques and vigilant postoperative care. A summary of our institution's practice recommendations is provided in Table 1.

**Table 1:** Considerations for managing spontaneous intracranial hemorrhage in patients with liver failure undergoing liver transplantation

Aspect	Considerations
Balancing bleeding risks with liver failure	Liver transplantation is the only definitive treatment
Coagulation and transfusion	Plasma exchange therapy in acute liver failure
	Viscoelastic test for managing coagulopathy
	Pro-hemostatic therapies: blood products and coagulation factors
	Platelet transfusion thresholds of at least 50x10^9^/L
	Maintaining fibrinogen levels above 150 mg/dL
Hemodynamic management	Systemic blood pressure management perioperatively
Neurosurgical Intervention	Fiberoptic ICP (intracranial pressure) monitor
	External ventricular drain
	Craniotomy
Other intervention	Neuroimaging follow up
	Non-invasive ICP monitoring: optic nerve sheath diameter
	Transplant surgical techniques: piggy back and portocaval shunt
	Dialysis perioperatively
Postoperative care	Platelet count follow up
	Venous thromboembolic prophylaxis

Limitations

The limitations of this study include its single-center design and small sample size, as well as the absence of a comparison cohort of patients with ICH who did not undergo LT. However, all patients with ICH in our series proceeded to transplantation, and each had an unpredictable neurologic trajectory at the time the decision to transplant was made. Therefore, no selection bias was introduced in determining which patients with ICH were offered LT under these circumstances.

## Conclusions

In conclusion, this article aims to guide clinicians through the complex decision-making process surrounding transplant eligibility in patients with stroke in the setting of liver failure. Our observations illustrate that with vigilant neurological assessment, meticulous hemostatic optimization and close interdisciplinary coordination, liver transplantation may remain a viable option for selected patients with spontaneous ICH. Further research into the mechanism of ICH in liver failure may yield valuable insights and help address the issue of organ futility in these high-risk scenarios. Continued accumulation of multicenter data and the development of a consensus-driven framework will be essential to improving outcomes and supporting clinicians faced with these challenging decisions.
